# One size fits all: Enhanced zero-shot text classification for patient listening on social media

**DOI:** 10.3389/frai.2024.1397470

**Published:** 2025-02-11

**Authors:** Veton Matoshi, Maria Carmela De Vuono, Roberto Gaspari, Mark Kröll, Michael Jantscher, Sara Lucia Nicolardi, Giuseppe Mazzola, Manuela Rauch, Vedran Sabol, Eileen Salhofer, Riccardo Mariani

**Affiliations:** ^1^Independent Researcher, Graz, Austria; ^2^Know Center Research GmbH, Graz, Austria; ^3^Chiesi Farmaceutici S.p.A, Parma, Italy

**Keywords:** patient-focused drug development, social media listening, patient’s perspective, patient centric, zero-shot classification, named entity recognition, relation extraction

## Abstract

Patient-focused drug development (PFDD) represents a transformative approach that is reshaping the pharmaceutical landscape by centering on patients throughout the drug development process. Recent advancements in Artificial Intelligence (AI), especially in Natural Language Processing (NLP), have enabled the analysis of vast social media datasets, also called Social Media Listening (SML), providing insights not only into patient perspectives but also into those of other interest groups such as caregivers. In this method study, we propose an NLP framework that—given a particular disease—is designed to extract pertinent information related to three primary research topics: identification of interest groups, understanding of challenges, and assessing treatments and support systems. Leveraging external resources like ontologies and employing various NLP techniques, particularly zero-shot text classification, the presented framework yields initial meaningful insights into these research topics with minimal annotation effort.

## 1 Motivation

Patient-focused drug development (PFDD) is revolutionizing the pharmaceutical industry, reorienting the drug development process to prioritize patient involvement (Perfetto et al., 2015). Beyond a mere shift in perspective, PFDD actively involves patients in decision-making processes, fostering a collaborative and patient-centric model. Clinical trial design stands out as a key arena influenced by PFDD. Integrating patient perspectives into trial protocols enhances the relevance and feasibility of studies, leading to improved recruitment and retention. This patient-driven design ensures that trials align with the practical experiences and preferences of participants. Moreover, PFDD refines benefit–risk assessments. Patient insights provide a nuanced understanding of a drug’s benefits, potential risks, and overall tolerability. Regulatory decisions, therefore, become more reflective of real-world implications, balancing efficacy with potential adverse effects (Sullivan, 2017; U.S. Food and Drug Administration, 2023).

While the advantages of PFDD are evident, reaching out to patients, caregivers, and other interest groups on a large scale is impractical. Social Media Listening (SML) emerges as a remedy, utilizing social media posts authored by patients and interest groups related to a specific disease (Limaye and Awani, 2018). These posts provide valuable insights into the daily challenges faced by individuals affected by the disease, complementing scientific findings by foregrounding the emotional burden experienced by patients and caregivers. SML allows for a direct comparison of perspectives between patients, caregivers, and representatives from the scientific or medical community or patient associations. This advantage has led to a growing interest in medical SML, particularly with advancements in Natural Language Processing (NLP) techniques (Convertino et al., 2018; Schmidt et al., 2022).

Regulatory authorities, including the United States Food and Drug Administration (FDA) and the European Medicines Agency (EMA), have increasingly recognized the value of patient experience data in drug development and regulatory decision-making. The use of social media in the context of PFDD has several considerations for regulatory authorities, particularly as outlined in FDA’s series of guidelines, which emphasize the importance of capturing experience data directly from patients, without third-party interpretation (FDA Draft Guidance 2018: Patient-Focused Drug Development: Methods to Identify What Is Important to Patients Guidance for Industry, Food and Drug Administration Staff, and Other Stakeholders, 2022).

For instance, the FDA’s guidance on PFDD emphasizes that Social Media Platforms can be used to gather patient perspectives on symptoms, disease impacts, and treatment experiences. These platforms include medical community blogs, crowdsourcing, and verified patient communities, allowing access to patient input during the drug development process.

Regulatory authorities provide a framework for ethical considerations, data privacy, and the representativeness of the data collected. They encourage stakeholders (such as pharmaceutical companies, patient advocacy groups, and academic researchers) to develop methodologies that are transparent, scientifically sound, and ethically compliant. By setting standards, regulators ensure that the data derived from social media can complement more traditional data sources and contribute meaningfully to product development and approval processes (FDA Draft Guidance 2018: Patient-Focused Drug Development: Collecting Comprehensive and Representative Input, 2020). When combined with other patient-focused data collection methods (like structured interviews, focus groups, or survey-based studies) SML adds depth and breadth to the data landscape (Cimiano et al., 2024).

The agency’s stance is that, whether automated or manually conducted, the methods used in SML must be demonstrated to be robust enough to support regulatory decision-making confidently. To achieve meaningful results at scale, two prerequisites must be met: well-defined research questions (U.S. Food and Drug Administration, 2023) and reliable techniques to automate the SML process. Research questions play an important role in defining the study’s scope, purpose, and relevance to healthcare challenges. In this work, we focus on identifying challenges and support systems for various interest groups, exploring three major research topics (RTs):

*Identification of Interest Groups (RT1)*: Who speaks about disease X? This task involves identifying various interest groups, including *patients*, *caregivers*, individuals from the *scientific or medical community*, and *patient associations*.

*Understanding of Challenges (RT2)*: What challenges does disease X pose? This task entails extracting burdens associated with the disease, such as *symptoms*, *drugs*, *treatments*, *side effects*, expressed *needs*, and the emotional burden of receiving a *diagnosis*.

*Assessing Treatments and Support Systems (RT3)*: Which treatment and support are available for people suffering from disease X? This task involves extracting information about *drugs*, *treatments*, and any other *support* mechanisms to alleviate challenges associated with the disease.

Selecting, adopting and applying the appropriate methods to address each research topic is of paramount importance. Answering each one of them requires a combination of sophisticated algorithmic methods from the field of NLP. While developing specific methods tailored to each research topic is desirable, it poses practical challenges due to the significant time and resource requirements. Therefore, we present an NLP framework for SML, incorporating four major NLP components: external knowledge bases, i.e., ontologies, few-shot text classification, zero-shot text classification also known as natural language inference, and question-answering. By leveraging state-of-the-art NLP methods, the presented framework aims to minimize development and annotation efforts while delivering robust results.

The research topics and the framework presented here are not meant to be limited to a specific disease and can be easily extended to other medical conditions as well. Idiopathic pulmonary fibrosis (IPF) was selected as the focus condition for our study, which utilizes NLP methods to extract relevant information regarding interest groups, challenges, and treatments, based on several strategic considerations aligned with the aims of FDA-led PFDD meetings (FDA-led Patient-Focused Drug Development (PFDD) Public Meetings, 2024). IPF is a chronic, progressive disease characterized by severe symptoms, such as shortness of breath, fatigue, and reduced physical capability, which significantly impact patients’ daily functioning. IPF has limited available treatments, with most therapies focusing on managing symptoms rather than halting disease progression. The FDA’s PFDD initiative prioritizes conditions that are chronic and symptomatic, particularly those that disrupt daily life, and diseases with few or insufficiently effective treatments, emphasizing the need for a deeper understanding of patient needs. Given its considerable impact on patients’ quality of life, IPF aligns well with these criteria, making it an appropriate candidate for a study that seeks to understand patient-reported experiences and challenges. The FDA’s PFDD initiative aims to bridge gaps between clinical trial data and real-world patient experiences, integrating patient perspectives into drug development and regulatory decisions (Chalasani et al., 2018).

The objective of this study is to present preliminary results and provide an overview, rather than conducting an in-depth analysis. The focus is on demonstrating the concept of utilizing an NLP pipeline to extract pertinent medical information from social media postings. This approach is instrumental in comprehending the challenges and support systems encountered by various interest groups.

This paper is organized as follows: Section 2 presents an overview of related work in the application of SML within the medical field. Section 3 details the criteria for data selection and describes the datasets as well as the annotation criteria utilized for training and evaluating the methodologies. In Section 4, we discuss the implementation of various methods aimed at addressing the RTs. Finally, Section 5 offers a discussion of the findings and a conclusion.

## 2 Related work

In the digital age, patients are becoming more active on the internet utilizing social media platforms to share their experiences, to engage in discussions about healthcare practices, to explore treatment options, to seek out healthcare professionals, and to express themselves openly (Hamm et al., 2013; Franklin et al., 2022). This proliferation of health-related content provides researchers and health professionals with a unique opportunity to tap into patient perspectives and therefore offers an innovative approach to collecting experience data from patients and other interest groups (Sivaratnam et al., 2022). In recent years, SML has gained significant traction for its potential to offer insights into specific aspects of the patient journey, among others, aiming for PFDD. The use of modern NLP approaches facilitates the exploration and analysis of large amounts of social media data and enables a wide range of applications.

Several works (Cook et al., 2019b; Koss et al., 2021; Schmidt et al., 2022) discuss the usage of SML leveraging Artificial Intelligence (AI) to support PFDD. Fisher et al. (2023) detects signals within social media posts of drug-related risk and harms and implements an early warning system by leveraging zero-shot classification on drug-related tweets. Kuntsche et al. (2023) monitors exposure and marketing of alcohol and other substances with high potential of abuse in digital media by using a zero-shot classification approach. Another field of SML focuses on patient burden analysis resulting from different diseases and their respective treatments. Sunkureddi et al. (2018) evaluates patients’ experience and their access to treatment for psoriatic arthritis. Wolffsohn et al. (2020) analyze how patients, suffering from age-related deterioration of vision (Presbyopia), write about their experience of symptoms and the impact on their life on related forums, blogs and news outlets. Utilizing GPT-3 for zero-shot question answering, Jiang et al. (2023) extracts COVID-19 related symptoms discussed by patients on Twitter.^1^ The study of Cook et al. (2019a) utilizes SML to shed light on patients’ experiences with dry eye disease, emphasizing previously unexplored aspects of patient experience within this indication. Karmalkar et al. (2023), amongst others, highlights the potential of modern NLP techniques for sentiment classification with zero-shot classification via natural language inference for social media posts on head and neck esophageal cancer.

It is evident that there is a growing trend toward efficiently extracting meaningful information from social media posts on biomedical topics using zero-shot methods (Kuntsche et al., 2023). These methods have been applied across various NLP tasks with minimal effort, including question-answering (Zhu et al., 2023), text-to-text generation (Gan et al., 2023), and sentiment classification (Manias et al., 2023).

Our work aligns with the growing trend of utilizing natural language inference models for various tasks. In this study, we aim to extend this approach to the text level. Unlike previous studies that primarily focus on text classification (Barker et al., 2021; Plaza-del-Arco et al., 2022), we apply natural language inference not only for classification but also for named entity recognition and relation extraction. By combining this with ontology-based named entity recognition and traditional supervised text classification methods, we provide a framework that enables SML in the medical domain without the need for extensive annotation and model training.

## 3 Data selection and processing

### 3.1 Data crawling

We collected posts between October 2021 and November 2023 from various social media sources using an enterprise-grade social listening tool. To capture mainly content related to the indication of interest, we established a set of keywords associated to IPF such as *Idiopathic Pulmonary Fibrosis, IPF, Pulmonary Fibrosis, Idiopathic Lung Fibrosis, anti-fibrotic agents* (*cf.* Supplementary Table 1), along with heuristics. Since users can post the same content multiple times under different URLs, we conducted deduplication to avoid bias.

**Figure 1:**
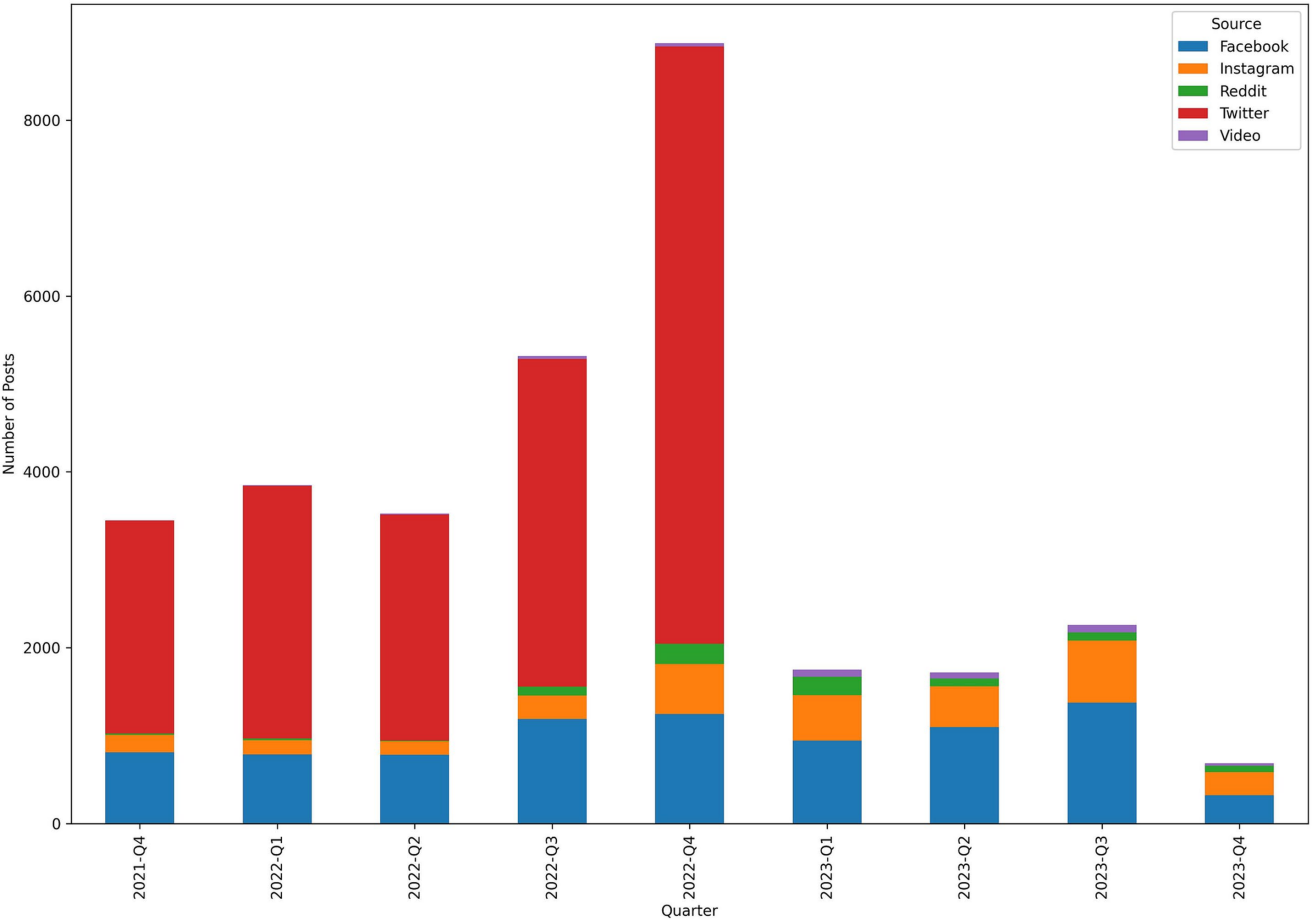
Distribution of posts for each quarter and source over the period of October 2021 to November 2023.

Figure 1 illustrates the number and distribution of posts from each source for each quarter.

Finally, before processing the content of the posts, we applied pseudonymization to the posts by replacing direct identifiers (El Emam et al., 2015). We employed spaCy^2^, which offers named entity recognition to detect names, and a token matcher to identify patterns resembling emails. To maintain readability, the original names were replaced with randomly generated equivalents using the Python library faker,^3^ a method also called *random substitution* (Mamede et al., 2016). Emails, on the other hand, were replaced by a generic placeholder, a method called *tagging* (Mamede et al., 2016).

**Figure 2:**
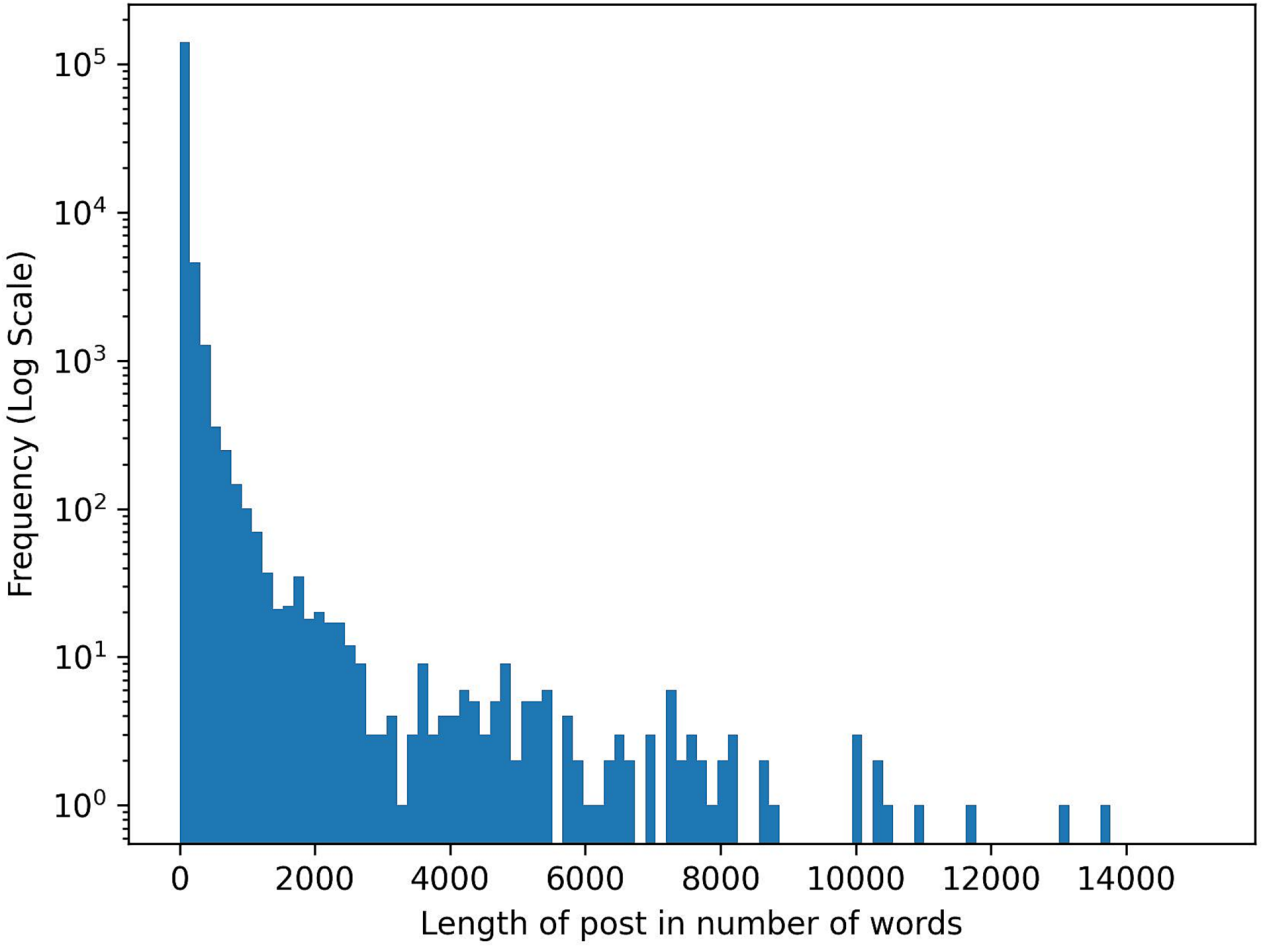
Post length histogram of crawled posts in number of words. As can be seen, the length between posts can vary considerably.

**Figure 3:**
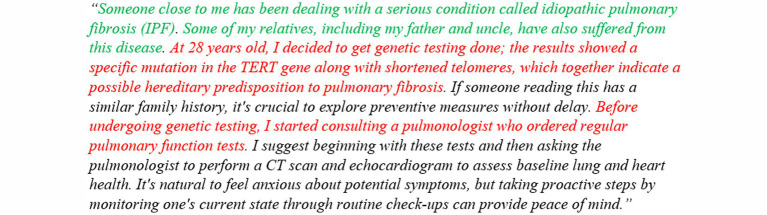
Example post demonstrating perspective shifts among different interest groups at the sentence level. This example illustrates the transition in perspectives between caregiver (highlighted in green) and patient (highlighted in red).

The length of the posts can vary considerably, as depicted in Figure 2. Many works on SML in the medical domain perform analyses at the post level (Cook et al., 2019c; Delestre-Levai et al., 2021). However, this approach presents challenges for fine-grained analyses and pinpointing specific information within longer texts – a challenge likely to intensify with the increasing number of long posts in the future. This difficulty is exemplified in tasks such as distinguishing between various interest groups, e.g., patients and caregivers (see Section 3.2.1). As demonstrated in Figure 3, a single post may encompass perspectives from multiple interest groups. Although the provided example is relatively concise, it illustrates the complexities that arise with longer blog posts, surpassing the typical brevity of Tweets.

While, in principle, such a change of perspectives can also happen on sentence level, the degree of variance is greater for posts. For the end user, it is crucial to precisely locate information within longer posts. Consequently, NLP methods and analyses were applied on the sentence level. In addition to the advantage of narrowing down the localization of specific information, a sentence-based approach facilitates the annotation and evaluation process because of the reduced ambiguity.

### 3.2 Datasets

To create datasets for training and evaluation purposes, we annotated a set of social media posts. The authors of the posts can have diverse backgrounds, ranging from medical professionals who post new medical findings to ordinary people without a medical background, such as patients or caregivers. These individuals express their daily experiences with their disease. Consequently, the style of writing can vary from colloquial to more formal, with scientific terminology. Nevertheless, the content remains medical in nature and necessitates a significant level of background knowledge to capture all medical concepts and their relationships.

To ensure precise annotations, comprehensive annotation guidelines were developed through empirical analysis of the data and in collaboration with drug development professionals experienced in PFDD. These guidelines underwent continuous refinement and were further enhanced by consulting various medical ontologies. Hiring dedicated annotators with a medical background was deemed unfeasible due to the high costs and the time required for training. However, this was not necessary for the following reasons: certain categories, such as interest group classification, did not require medical expertise, and for the remaining categories, the annotation guidelines and access to pharmaceutical experts provided sufficient support.

Two data scientists with advanced English skills and prior experience in data annotation were selected to perform the annotations after thorough training on the guidelines. The open-source tool INCEpTION^4^ was used for conducting the annotations, which were performed at the sentence level rather than at the post level to ensure greater clarity and enable more precise analyses in subsequent stages. Drug development professionals were available for consultation on medical-specific questions, and all annotations were ultimately cross-checked by these experts to ensure accuracy and consistency.

The annotation guidelines provided detailed instructions for annotating content in the following categories: interest group, (medical) named entity, and relation. In the following, we briefly present each category and the respective dataset.

#### 3.2.1 Interest group classification dataset

Within the interest group category, we establish distinctions among sentences originating from a *caregiver*, a *patient*, a *patient’s association*, the *scientific or medical community*, or *other* groups. These categories are mutually exclusive. Defining patients and caregivers is a crucial task, and their definitions can vary significantly in medical literature. For instance, Delestre-Levai et al. (2021) describe patients’ and caregivers’ posts as follows: *“First-person mentions of the disease or treatment were automatically classified as patient conversations, whereas third person references to the disease experiences of a child, parent or family member, or their care were identified as caregiver conversations.”* Cook et al. (2019a) follow a similar scheme. During the data analysis, it was discovered that many instances of first-person mentions of a disease or treatment do not fit the criteria for patients’ posts. As a result, we developed more restrictive definitions:

*Caregiver (CG)*: The author of the sentence either explicitly or implicitly indicates a close relationship with a sick person or a patient. A specific indication that the person is taking care of that sick person or patient is not required. Caregivers typically include family members, friends, or acquaintances. Caregiver posts are written in the first person, thus conveying personal information or experiences. Examples are: *“My loved one has been diagnosed with idiopathic pulmonary fibrosis, which is considered terminal, making long-distance travel increasingly difficult for us.”; “Since June 10th, my mother has been extremely fatigued, but unfortunately, we have been unable to access suitable medical care within our country.”*

*Patient (PA)*: The author of the sentence either explicitly or implicitly identifies themselves as a patient or a sick person. To classify a post as originating from a patient, it must contain explicit mentions of a medical condition. Patient posts are written in the first person, thus conveying personal information or experiences. Examples are: *“As someone who lives with disabilities, our community has recently faced a devastating natural disaster that left us struggling to cope - unfortunately, we received little support from local organizations, which only highlights the pressing need for accessible home modifications.”; “After battling two episodes of pneumonia, I’m left with irreparable lung damage from my compromised immune system, which has led to the development of both emphysema and pulmonary fibrosis.”*

Besides that, we added two more interest groups, namely:

*Patient’s association (PAA)*: The sentence pertains to events, organizations, workshops, etc., designed for patients and their caregivers. The exception to this rule is for scientific conferences or scientific workshops. Examples are: *“I recently contributed to a tribute fund for {ASSOCIATION}. This effort was initiated by our community living with Pulmonary Fibrosis, who came together to create a user-friendly online platform (#{PROJECT}, accessible at {URL}) for easily navigating relevant data.”*

*Scientific or medical community (SC)*: The sentence pertains to scientific or medical papers, studies, clinical trials, findings, as well as events like conferences or workshops. Examples are: *“A leading research institution advocates for pulmonary fibrosis treatment by enhancing the recycling process of TGF-beta receptor type I [1]. This organization has made significant contributions to tissue repair and wound healing across* var*ious organs.”; “I am pleased to share that my abstract: {TITLE} has been selected for an oral presentation at our organization’s annual research conference later this year.”*

*Other (O)*: Any sentence that cannot be categorized into the four previously listed categories falls into this miscellaneous group. Examples are: *“I’m afraid this is not the situation we are dealing with.”; “We’d love to relocate, but it’s just not feasible for us right now.”; “But not this.”*

We annotated 1323 sentences (*cf.* Table 1 for more detailed information) which were used for both training and evaluating a text classification model.

**Table 1:** Distribution of labels for each interest group in the annotated dataset, including train-validation-test splits.

Label	Num. examples (Train/Validation/Test)
Caregiver	144 (87/29/28)
Patient	230 (138/46/46)
Patient’s association	104 (62/21/21)
Scientific or medical community	132 (79/27/26)
Other	713 (427/142/144)
Total	1,323 (793/265/265)

#### 3.2.2 Named entity recognition dataset

We distinguish between *primary* and *secondary biomedical named entities*. The category of primary biomedical named entities includes:

*Disease (DI)*: Names and abbreviations of diseases, such as *pulmonary fibrosis, IPF* etc.

*Drug (DR)*: Names of chemicals and drugs, including brand names, such as *pirfenidone, nintedanib,* etc.

*Symptom (SY)*: Names of symptoms associated with a disease.

*Treatment (TR)*: Names of medical methods, techniques or devices that are used to treat diseases or symptoms. The key difference to *drug* is that treatments are not chemical.

These categories are mutually exclusive, meaning they do not overlap. Distinguishing between these primary biomedical named entities is generally clear, except for the distinction between *disease* and *symptom*, as suggested by the overlapping entries in ontologies (see Section 4.2).

Under the term *secondary biomedical named entity*, we cover those biomedical named entities whose identification relies on the previous primary biomedical named entities. Often, the identification of secondary biomedical named entities requires capturing the interaction between the primary biomedical named entities:

*Diagnosis*: Any disease or symptom that is stated to be diagnosed or detected is an instance of *diagnosis*. Therefore, to identify a diagnosis, you need to be able to identify diseases or symptoms first.

*Misdiagnosis*: Any disease or symptom that is stated to be misdiagnosed or was missed during examination is an instance of *misdiagnosis*. Therefore, to identify a misdiagnosis, you need to be able to identify diseases or symptoms first.

While not directly tied to the primary task of extracting biomedical named entities, this category also encompasses the extraction of time-related mentions. Analyzing time-related mentions and their correlation with the previously mentioned biomedical named entities yields additional insights into the challenges and support experienced by patients and caregivers. For instance, the burden of living with a disease for an extended period seems to be greater than dealing with a recently acquired condition. The labels for time-related mentions are taken directly from the dataset of Almasian et al. (2022) and are:

*Duration (DU)*: Expressions of any durations, such as *3 months, 2 weeks, days* etc.

*Date (DT)*: Expressions of a date, such as *yesterday, tomorrow, November 3rd, 2023*.

*Time (T)*: Expression of a time of day, such as *3 o’ clock, 7 pm* etc.

*Set (S)*: Expressions that describe some sort of frequency, such as *daily, weekly* etc.

As outlined in Section 4.2, for named entity recognition we use external ontologies as well as an already fine-tuned model. Hence, the dataset was used exclusively for testing purposes (*cf.* Table 2).

**Table 2:** Distribution of labels for each named entity in the annotated dataset.

Label	Num. examples
Date	91
Disease	297
Drug	106
Duration	49
Set	19
Symptom	149
Time	4
Treatment	67
Total	782

This dataset was exclusively used for testing purposes; hence no train-validation-test splits are provided.

#### 3.2.3 Relation extraction dataset

The relationships between extracted medical concepts and time mentions must be identified. In line with the research topics at hand, we have established the following relations:

*Ameliorates (AM)*: This relation exists between a treatment (including drugs) and a disease/symptom when the use of the treatment leads to the improvement of symptoms caused by the disease. It implies a therapeutic interaction where the treatment positively impacts the disease’s progression or alleviates the severity of its symptoms, contributing to the patient’s relief or recovery. Typical trigger words are *improve, ameliorates, amelioration, decrease, reduce* etc.

*Creates (CR)*: This relation is established when one biomedical named entities (such as a *disease*, *treatment*, or *drug*) directly leads to the occurrence of another medical condition or symptom. For example, this relation holds if a disease causes a specific symptom, or conversely, if a symptom is indicative of a particular disease. Similarly, it applies when a treatment or drug results in the emergence of a new disease or symptom. The *creates* relation signifies a causal or contributory link, where the presence or administration of one entity is responsible for the genesis of another condition or symptom. While we drew inspiration from the existing definition of causality by Dunietz et al. (2017), we opted for a more restrictive definition. For example, according to our annotation guidelines, temporal causation, as indicated by conjunctions such as *after*, does not qualify for the relation *creates*. Also, expressions such as *IPF cough* do not qualify as instances of the relation *IPF #causes# cough*, since an explicit cue word indicating a causation is missing.

*Diagnoses (DG)*: This relation specifically exists between a treatment or diagnostic method and a disease or symptom. It is applicable when a particular treatment or diagnostic technique is explicitly used to detect or diagnose a disease or symptom. The diagnoses-relation implies that the method or treatment in question is instrumental in identifying the presence, nature, or severity of a particular medical condition or symptom. This relation is central to clinical practice, as it connects diagnostic procedures directly with the medical conditions they are intended to identify or confirm.

*Exacerbates (EX)*: This relation is used to describe a scenario where one entity X, a disease, symptom, treatment, or drug, causes a worsening or aggravation of another entity, typically a medical condition or symptom. This relation is specifically applied when there is an explicit description of the worsening effect. It encompasses cases where X leads to an increase or intensification of Y. The *exacerbates*-relation also subsumes instances where one entity X inadvertently promotes the progression or severity of a disease, like the growth of a tumor. The key aspect of this relation is the explicit and discernible intensification of a medical condition or symptom due to the influence of another factor. Typical trigger words are *exacerbate, worsen, worse* etc.

*Is associated with (IAW)*: There are cases where it is very likely that one entity X causes another entity Y, nevertheless the linguistic information provided is too weak or ambiguous to draw this conclusion. These cases, where there is a lack of an explicit connective or a connective that is too general (expressions such as *is associated with, is connected to, is linked* to etc.), will be subsumed under the relation *is associated with*.

*Is used for (IUF)*: This term encompasses instances where an entity X is identified as a treatment for entity Y. In such cases, no explicit information about the efficacy of the treatment is provided; that means, it remains unspecified whether the treatment ameliorates or exacerbates a disease or symptom. Common indicators include phrases like *is used as a treatment for* or *is a treatment for*. Sometimes, direct connectives may be absent, necessitating reliance on context. Here, if a treatment is recognized and the sentence contextually links the treatment with a disease or symptom, it is inferred that the treatment pertains to the disease/symptom. This inference approach is deemed appropriate as it does not speculate on the treatment’s effectiveness or outcome.

*Is time of (ITO)*: This designation encompasses instances where a specific point in time marks the onset of a disease, symptom, or similar event. While recognizing that a single time-relation might oversimplify the complexity inherent in time-relation extraction, as indicated by the extensive range of possible time-relations identified in Gumiel et al. (2022), this approach nonetheless proves adequate for initial results.

Almost all relations are unidirectional, meaning that swapping the positions of the nodes in a triple change the meaning of the relation: *cough #causes# IPF* ≠ *IPF #causes# cough*. The only exception to this rule is the relation *is associated with*, where swapping the positions of the nodes does not impact the semantics of the resulting triple: *cough #is associated with# IPF = IPF #is associated with# cough*.

The semantic distinction between some relations is subtle. For example, the relations *creates* and *exacerbates* can be considered subclasses of the broader relation *is associated with*. A similar relationship holds between *ameliorates* and *is used for*, with the former being a subclass of the latter. In the subsequent section we will refer to the *is associated with* and *is used for* relations as macro-relations and to their subclasses as micro-relations.

As detailed in Section 3.2.3, relation extraction is deployed after named entity recognition, since the listed relations in the pipeline can only exist between pre-extracted concepts. Therefore, the relation extraction dataset developed for this study follows the structure of the SemEval-2010 Task 8 (Hendrickx et al., 2009): For each sentence, we provide information about two concepts present in the sentence. The dataset’s task is to extract the relation and determine the direction of the relation between these two concepts. To simulate real-world scenarios, the dataset also contains sentences with concepts that do not exhibit any relations.

It is essential to emphasize that the annotations of relations and, consequently, relation extraction aims to identify relations as they are stated in the text and not as they exist in reality. This becomes particularly significant when considering the diversity of social media post authors, the majority of whom lack a medical background and may provide scientifically untenable information.

As outlined in Section 4.3, for relation extraction we use natural language inference models in combination with heuristics and ontologies. Hence, the dataset was used exclusively for testing purposes (*cf.* Table 3).

**Table 3:** Distribution of labels for each relation in the annotated dataset.

Label	Num. examples
Ameliorates	11
Creates	41
Diagnoses	32
Exacerbates	7
Is associated with	49
Is time of	53
Is used for	66
NoRel	104
Total	363

This dataset was exclusively used for testing purposes; hence no train-test splits are provided. The term “NoRels” refers to instances where named entities are identified in the document, yet no relationships exist between them.

## 4 Methods

This section outlines and provides details on the NLP tasks that need to be performed to address the research topics.

*Text Classification* is a fundamental task in NLP where a label *y 𝜖 Y* is assigned to a given text document *X = {x1, …, x_n_}*. The label *y* represents a category or class from a predefined set *Y*, and the document *X* consists of *m* words or tokens. This task involves determining the overall theme or category of the text, such as sentiment analysis, topic labeling, or intent detection. In this domain, we distinguish between zero-shot and few-shot text classification. Zero-shot classification refers to the model’s ability to correctly classify text into categories it has never seen during training, relying on its understanding of language and category descriptions. Few-shot classification, on the other hand, involves training the model on a very small amount of labeled data for each category, testing the model’s ability to generalize from minimal examples.

*Named Entity Recognition* is a typical sequence labeling task that assigns an entity type *y 𝜖 Y* to each word *x* in a given sentence *X = {x1, …, x_n_}*, where *Y* denotes the set of entity labels and *n* denotes the index of the given sentence in a list of sentences. Named entity recognition aims to identify and classify named entities in text into predefined categories such as names of persons, organizations, locations, expressions of times, quantities, monetary values, percentages, etc. In this paper, the terms *concept* and *named entity* will be used interchangeably.

*Natural Language Inference* is a task where the goal is to determine the logical relationship between a pair of sentences, namely a premise *P* and a hypothesis *H*. The objective is to assign a label *y 𝜖 Y*, typically including labels such as *entailment*, *contradiction*, or *neutral*, indicating whether the hypothesis *H* is true (*entailment*), *false* (*contradiction*), or undetermined (*neutral*) given the premise P. Natural language inference plays a critical role in understanding and interpreting the meaning of sentences in context. Yin et al. (2019) demonstrate that natural language inference approaches excel in zero-shot text classification tasks, a strength leveraged in the design of this method.

*Question-Answering* is a task where the system generates an answer *A* based on a given question *Q* and an optional context *C*. The context *C* can be a passage of text or a larger corpus from which the answer needs to be extracted or inferred. The task involves understanding the question *Q*, processing the relevant context *C*, and producing a concise and accurate answer *A*. Question-answering systems can vary from simple factoid question answering to more complex ones requiring reasoning over multiple pieces of information.

For the evaluation, we used the following standard metrics: precision, recall, and F1-score, each presented in both their macro-and weighted-average forms.

**Figure 4:**
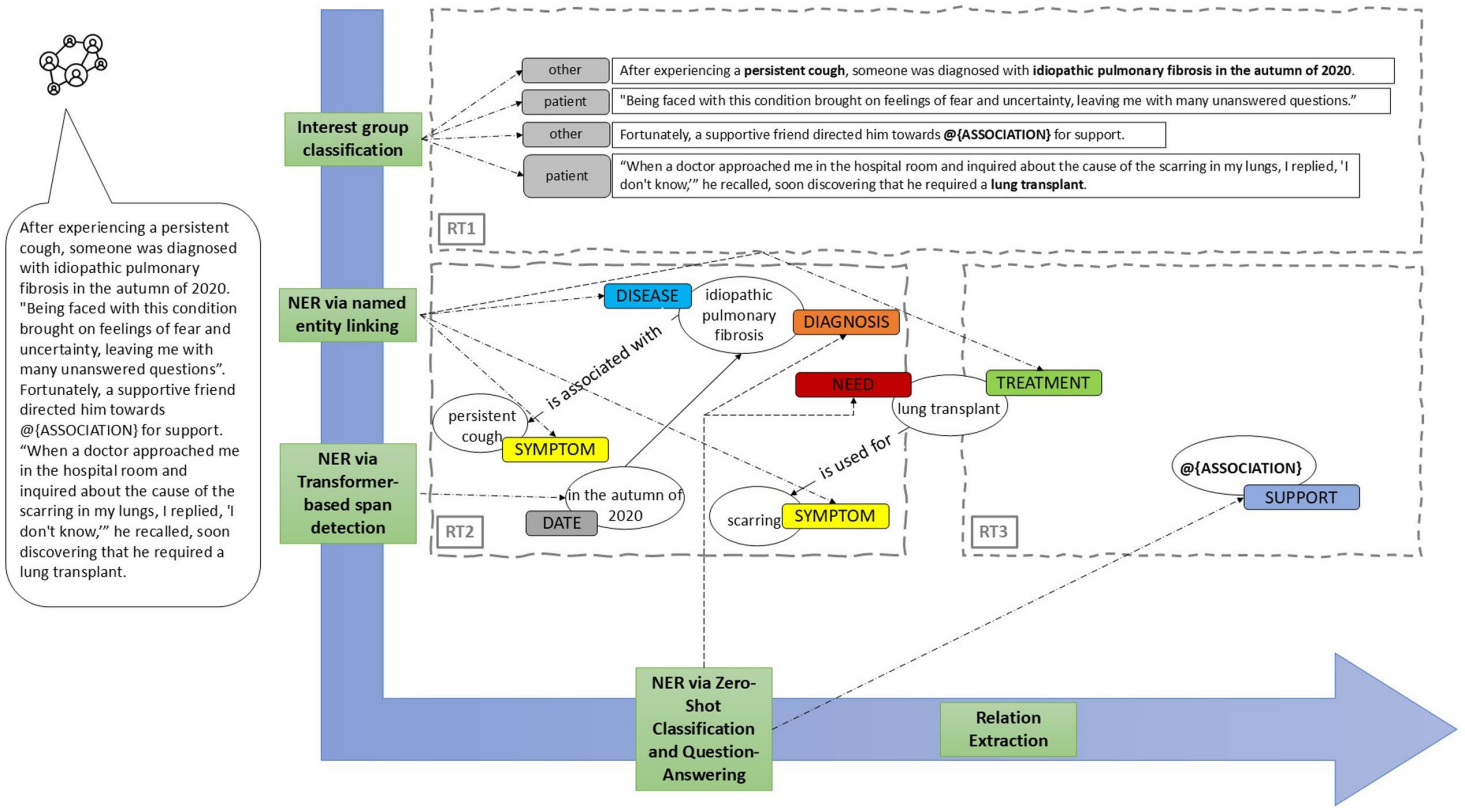
For RT1, sentences are classified according to predefined interest groups using text classification, specifically employing the SetFit model as detailed in Section 4.1. For RT2 and RT3, multiple NLP methods are utilized. Named entity recognition is used to extract entities, which are subsequently classified based on their nature: “challenges” (e.g., the symptom “persistent cough”), “support” (e.g., the name of the association “@{ASSOCIATION}”), or occasionally both (e.g., “lung transplant,” which may be considered a burden when expressed as a need but a form of support when mentioned as a treatment for medical conditions). Biomedical named entities are identified using ontologies, while more abstract entities such as “need” or “support” are discerned via zero-shot classification and question-answering techniques. To elucidate the relationships between named entities (e.g., “lung transplant #is used for# scarring”), we apply zero-shot classification in tandem with heuristic methods.

Figure 4 presents a high-level overview of the NLP methods applied to each research topic.

### 4.1 Interest group classification

Since the interest group dataset was relatively small, we adopted a few-shot learning approach. We leveraged Sentence Transformer Fine-tuning, a fast and lightweight solution proposed by Tunstall et al. (2022). This method fine-tunes a pre-trained Sentence Transformer (Reimers and Gurevych, 2019), using a small number of text pairs in a contrastive Siamese manner. The resulting model is employed to generate rich text embeddings, which, in turn, are used to train a classification head. We selected the Sentence Transformer model *sentence-transformers/all-mpnet-base-v2*^5^ and trained it with the following hyperparameters: learning rate: 5e-05, batch size: 16, number of iterations: 1, and number of epochs: 1. The results on the test set are presented in Table 4.

**Table 4:** Results of interest group classification on the test set.

	PA	CG	SC	PAA	O	ACC	MACRO	WEIGHTED
Precision	90.24	85.19	92	90.48	91.39	90.57	89.86	90.52
Recall	80.43	82.14	88.46	90.48	95.83	90.57	87.47	90.57
F1	85.06	83.64	90.2	90.48	93.56	90.57	88.59	90.46

For all classes, very good results are achieved despite the relatively small training dataset.

**Figure 5:**
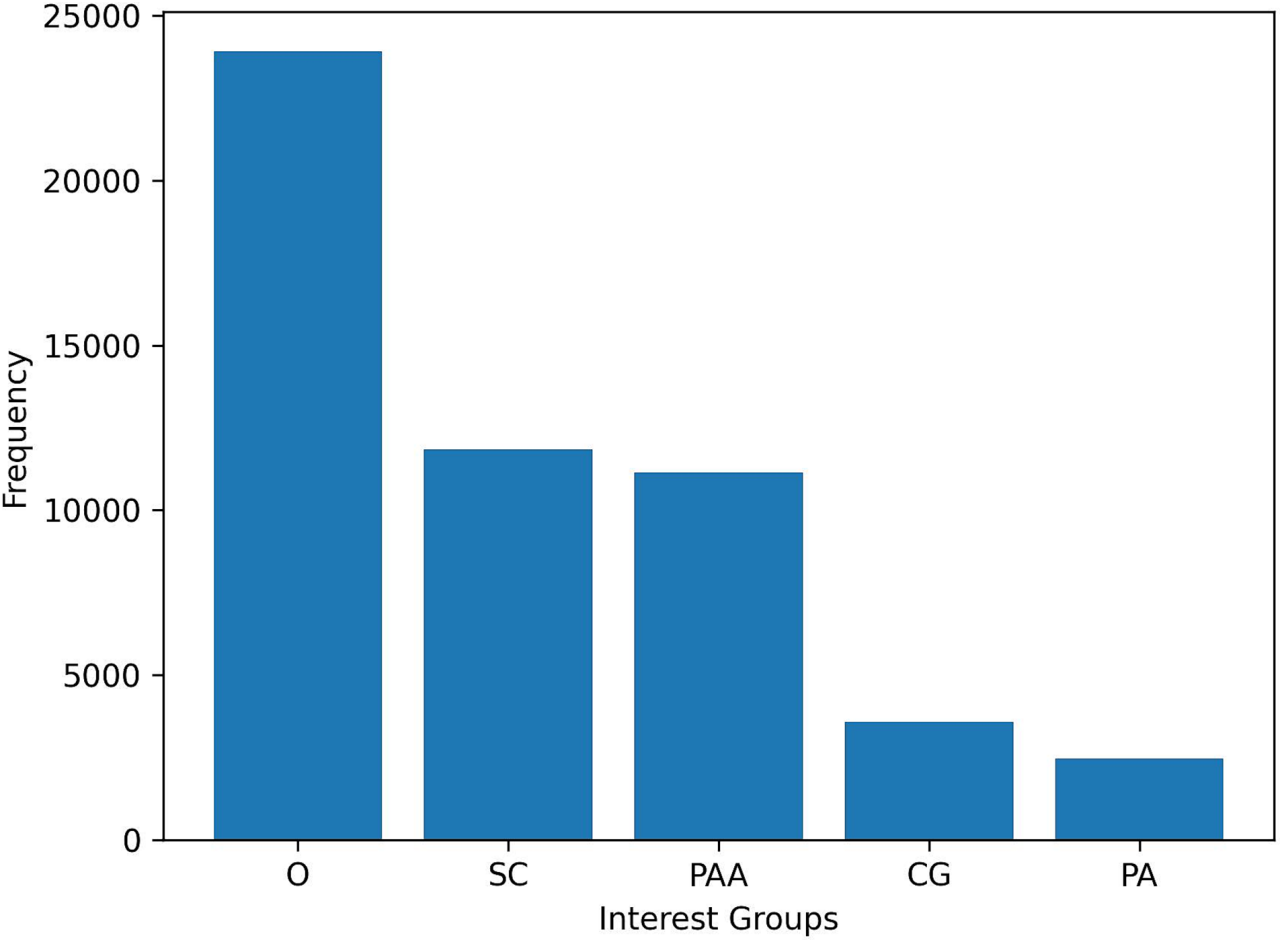
Distribution of interest groups in the dataset.

Despite the relatively small number of training examples, we have successfully trained a classifier that is capable of accurately distinguishing between different interest groups. The model was applied at the sentence level across the entire dataset of social media posts that were crawled. Subsequently, we assigned labels to each sentence, resulting in a multi-label output at the post level. Figure 5 depicts the uneven distribution among the interest groups: notably, caregivers and, more significantly, patients represent the smallest groups. This discrepancy is noteworthy given that in most other studies we have reviewed (Cook et al., 2019c; Delestre-Levai et al., 2021), the analysis is typically restricted to two interest groups, namely *patient* and *caregiver*. The findings suggest that incorporating at least one additional category, such as *other*, to classify posts that do not fit into the *patient* or *caregiver* categories can yield more reliable and robust results.

### 4.2 Named entity recognition

As described in Section 3.2.2, the named entity recognition dataset encompasses an extensive set of named entities. Each type of named entity requires different strategies, which will be explored in the subsequent subsections.

#### 4.2.1 Named entity recognition via named entity linking

Named entity linking is the process of identifying and linking entities mentioned in text to corresponding entities in a knowledge base, thereby providing context and unambiguous identification of these entities. This technique finds application in various fields, including the biomedical domain (French and McInnes, 2023). The specific named entities we aim to extract using this method, which we refer to as primary biomedical named entities, include *disease*, *symptom*, *drug*, *treatment*.

As the set of concepts, we aimed to detect is extensive, annotating a large dataset for training a named entity recognition model would have been a laborious task. Consequently, we chose to leverage ontologies, which have proven to be a promising approach (Yoon et al., 2022). This was achieved by utilizing spaCy’s matcher pipeline^6^ to process thousands of entries from various ontologies (*cf.* Supplementary Table 2) and identify their presence in documents. Most of these ontologies are medical in nature, except for the Wikidata which, in absence of dedicated ontologies, we used to compile a list of terms for the concept *treatment*: We retrieved all terms classified under the superclasses *therapy* and *medical procedure*. Additionally, we excluded entries classified as *health assessment*, *invasive test*, *medical diagnosis*, and *medical test*. This exclusion was necessary as these categories are more likely to be associated with diagnostic methods rather than treatments. Upon manual review, the compiled list for treatments was found to produce favorable outcomes. Employing ontologies was especially effective, primarily because medical ontologies tend to consist of unambiguous terms. Nevertheless, to minimize the occurrence of false positives, we implemented several filtering mechanisms, including:

- Ignoring all matches that were part of spans belonging to the entity class ORGANIZATION. This step was considered crucial, as many foundations and organizations include disease names in their titles, as seen with the *Canadian Pulmonary Fibrosis Foundation*.
- Disregarding matches whose phrase heads were not nouns or proper names.
- During certain iterations, we encountered ontologies that contained entries with overly broad semantics, such as *group*, *role*, and *application* within the DrOn ontology. To address this, we compiled blacklists for each ontology to exclude such ambiguous terms that could result in false positives. This approach yielded satisfactory initial results; however, future efforts will necessitate more comprehensive ontology curation and the integration of more sophisticated techniques for entity linking.
- Distinguishing between diseases and symptoms is challenging, as suggested by the overlapping entries in disease and symptom ontologies. Considering that many of the social media posts we analyzed were written by non-experts, it was very difficult to draw a clear distinction between diseases and symptoms. The classifications provided in these texts are often unreliable. Therefore, after reviewing the overlaps, we decided to treat each overlap as a symptom.

Table 5 (Panel A) showcases the results of the named entity recognition pipeline applied to the named entity recognition dataset. As anticipated, the precision score outperforms the recall score, a pattern most noticeable in the extraction of symptoms and treatments. Future improvements in named entity recognition, such as thorough ontology curation or advanced training of a named entity recognition model, are crucial. This is especially important since the effectiveness of relation extraction (see Section 4.3) hinges on the named entity recognition performance. Currently, the results are adequate to operationalize the pipeline and to gain initial insights.

**Table 5:** Results of named entity recognition of primary medical named entities on the named entity recognition dataset (A) and results of named entity recognition of time-related mentions on the named entity recognition dataset (B).

(A)							
	DI	DR	SY	TR	Micro	Macro	Weighted
Precision	69.57	61.32	75	77.14	69.14	70.76	70.28
Recall	70.03	61.32	36.24	40.3	57.19	51.97	57.19
F1	69.8	61.32	48.87	52.94	62.6	58.23	61.48

(B)							
	DU	DT	T	S	Micro	Macro	Weighted
Precision	72.97	91.49	100	62.5	81.91	81.74	82.75
Recall	55.1	47.25	50	26.32	47.24	44.67	47.24
F1	62.79	62.32	66.67	37.04	59.92	57.2	59.62

This approach facilitates preliminary conclusions concerning RT2 and RT3. For example, by analyzing symptoms that co-occur with IPF or its synonyms within the same sentence, we can derive initial insights into the potential health-related challenges faced by patients (*cf.* Supplementary Figure 1). In a similar manner, a co-occurrence analysis of treatments allows us to make preliminary assumptions regarding the support provided to patients and their caregivers (*cf.* Supplementary Figure 2). For comparable overviews pertaining to the other category *disease*, please see Supplementary Figure 3.

However, mere co-occurrence is not sufficient to draw meaningful conclusions, which is why we implemented relation extraction (see Section 4.3).

#### 4.2.2 Named entity recognition via transformer-based span detection

For the extraction of time-related mentions, we utilized the BERT-based named entity recognition model for time mention extraction described by Almasian et al. (2022)^7^ the results of which are depicted in Table 5 (Panel B). A preliminary error analysis revealed discrepancies between our definitions of certain time concepts and those used to annotate the dataset in the study by Almasian et al. (2022). For instance, the adverb *now* was consistently tagged as a *date* instance in our test dataset: We deemed this significant for identifying the onset of diseases or symptoms. The utility of time-related mentions becomes particularly evident when integrated with biomedical named entities for relation extraction, as discussed in Section 4.3.

#### 4.2.3 Named entity recognition via zero-shot classification and question-answering

We aim to extract new information dynamically, beyond the predefined sets of named entities used in previous methods. This objective includes understanding the general needs expressed by different interest groups and the support they receive, which extends past mere medical concept extraction. This poses a challenge due to the abstract nature of these concepts, as they are not limited to a specific domain. For instance, needs may be social or psychological, and the same variability applies to support types. To address this complexity, we utilize zero-shot text classification and question-answering models, leveraging their capabilities to decipher abstract and domain-agnostic information.

**Figure 6:**
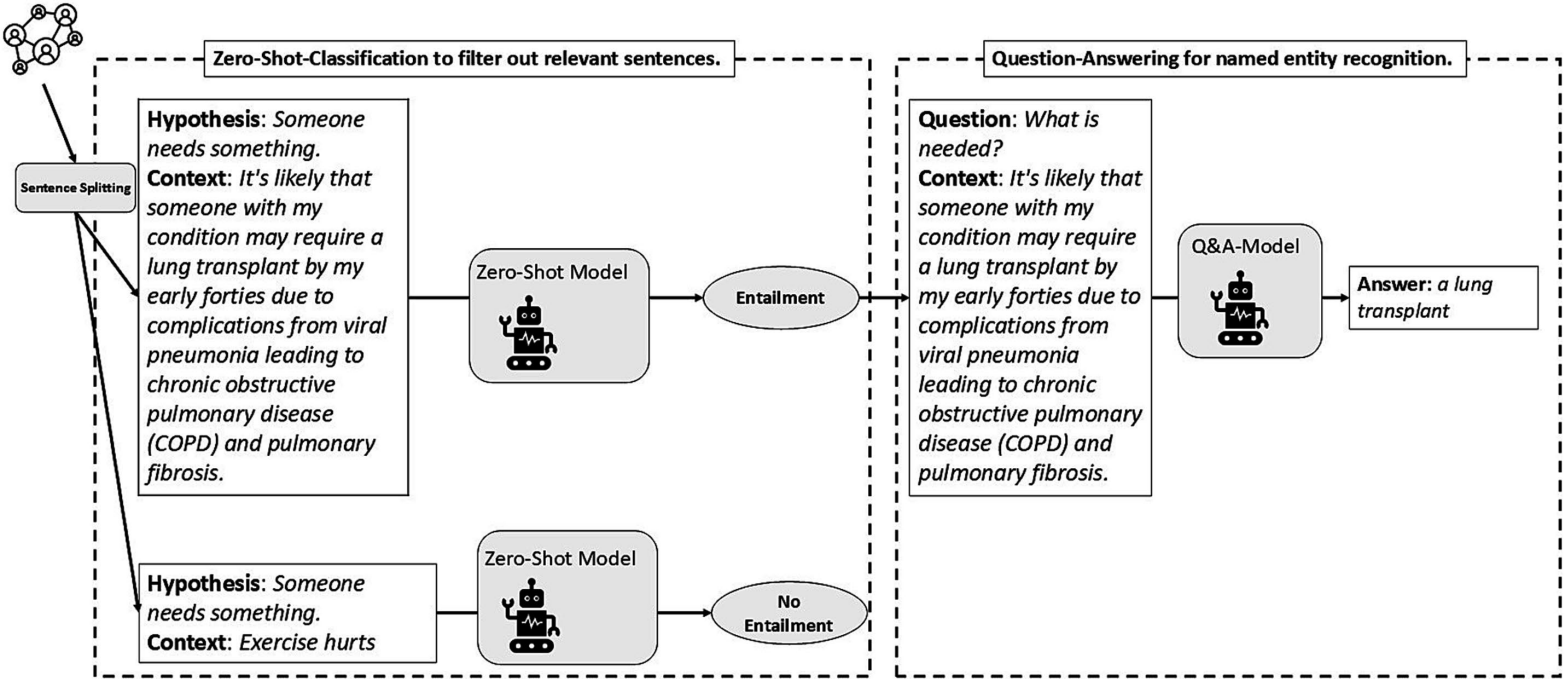
Illustration of the deployment of zero-shot text classification and question-answering models for named entity recognition.

Figure 6 illustrates the deployment of zero-shot text classification and question-answering models. Initially, a post is divided into sentences. Then, based on the target named entity, each sentence (i.e., the context) along with several predefined hypotheses is processed by a zero-shot text classification model to determine which sentences might contain relevant information. For instance, the hypothesis “Someone needs something.” is used to classify sentences according to whether they express a need. The model generates a confidence score ranging from 0 to 1 for each hypothesis. If the confidence score for a particular sentence exceeds a predefined threshold—determined through trial and error—it is inferred that the sentence entails the hypothesis; in other words, the hypothesis is validated. For example, the first sentence in Figure 6 indicates a need, the second sentence does not express a need. In cases of entailment, the first sentence then serves as context for a question-answering model. Corresponding to each hypothesis, one or more questions are formulated to extract more specific information within the sentence. In the provided example, by inputting the first sentence and the question “What is needed?” Into the question-answering model, *a lung transplant* is extracted as the explicit need expressed in that sentence. In a similar vein, information regarding support can also be extracted.

**Figure 7:**
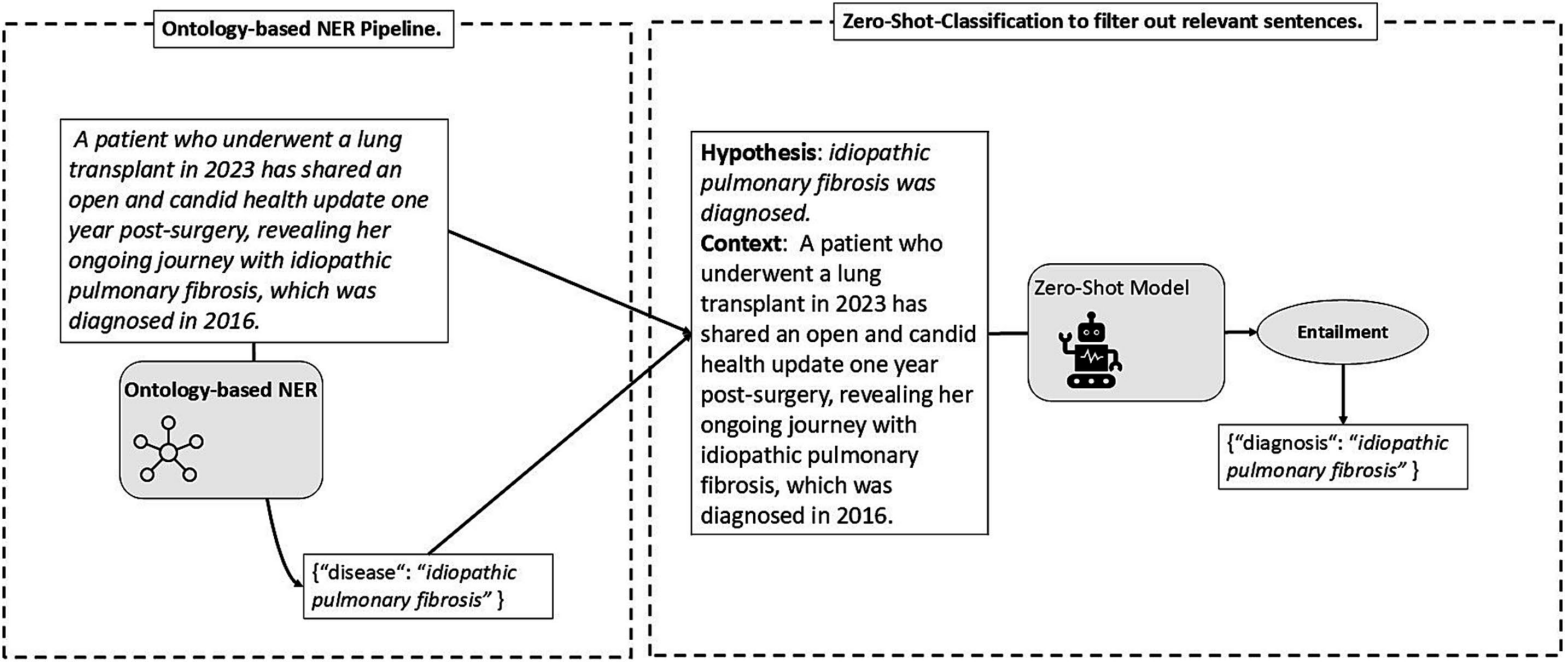
Illustration of the deployment of ontologies and zero-shot text classification for named entity recognition.

While natural language inference models are effective in zero-shot classification tasks, their performance varies across domains. For instance, domain-specific hypotheses like “Someone talks about a diagnosis.” may not be well-handled by models fine-tuned on non-medical datasets. However, incorporating domain knowledge to some extent is feasible by means of ontologies, as depicted in Figure 7. According to Section 3.2.2, any disease or symptom identified or detected qualifies as an instance of diagnosis. To pinpoint instances of diagnosis, an effective strategy involves first identifying any symptom or disease and then evaluating, within the given context, if it is a diagnosis. Ontologies facilitate the extraction of diseases and symptoms, which, along with the full context (i.e., the sentence), enables the crafting of hypothesis-context pairs for truth value assessment. For example, upon identifying idiopathic pulmonary fibrosis as a disease instance, we can formulate the hypothesis “idiopathic pulmonary fibrosis was diagnosed.” or “idiopathic pulmonary fibrosis was detected.” Given the context, zero-shot classification allows us to validate this hypothesis, thus categorizing idiopathic pulmonary fibrosis as an instance of diagnosis. In a similar vein, information regarding *misdiagnosis* can also be extracted. An overview of the hypotheses used, i.e., templates, along with the pertinent questions, is provided in Supplementary Table 3.

For zero-shot-classification we used a DeBERTa-based model,^8^ and for question-answering a RoBERTa-based model.^9^

We conducted a qualitative analysis of randomly selected samples from each category, namely *need*, *diagnosis*, *misdiagnosis*, and *support*. This analysis revealed that the results are adequate for obtaining initial insights. Table 6 showcases example sentences for extracted instances of *need*, *diagnosis*, and *misdiagnosis*, serving as representatives of potential challenges in RT2. It also includes instances of *support* pertinent to addressing RT3.

**Table 6:** Example sentences illustrating the extraction of concepts: need, diagnosis, misdiagnosis, and support.

Category	Sample sentence
Need	*She sought access to* **oral contraceptives** *but they were not readily obtainable for her*.
Need	*Currently hospitalized in Oklahoma, our efforts focus on securing a transfer to a university hospital for* **a second opinion** *with the aim of enhancing her quality of life*.
Need	*A local resident in need of* **a lung transplant** *is struggling to survive due to severe complications arising from COVID-19 infection, alongside a debilitating condition known as pulmonary fibrosis that severely impacts lung function*.
Need	*Idiopathic pulmonary fibrosis (#IPF), a debilitating lung condition, urgently requires increased* **awareness** *and understanding among healthcare professionals and the general public alike.{URL}*
Need	**Efforts to increase public knowledge and financial support** *are vital for advancing research into pulmonary fibrosis and discovering effective treatments.*
Diagnosis	*I previously donated platelets, but my health took a turn in 2022 when I received a diagnosis of* **pulmonary fibrosis**, *forcing me to adjust my treatment plan significantly and incorporate multiple medications, including immunosuppressant therapies*.
Diagnosis	*A recent study presents a tailored deep convolutional neural network design, known as Fibrosis-Net, aimed at predicting* **pulmonary fibrosis** *progression from chest computed tomography (CT) images*.
Diagnosis	*Lung function tests showed poor results and imaging scans revealed that I have developed* **pulmonary fibrosis** *due to COVID-19 complications*.
Misdiagnosis	*An acquaintance of mine passed away from* **pulmonary fibrosis** *which remained unacknowledged by his physician for several years because he attributed his sudden, severe difficulty breathing to her weight*.
Misdiagnosis	*A woman I know has been living with undiagnosed* **Rheumatoid Arthritis** *for more than a decade*.
Misdiagnosis	*To clarify: A misattribution of diagnosis can occur when a condition such as* **COPD** *is inaccurately described, despite accurate investigative procedures being mentioned* *(including pulmonary function tests, blood work, chest X-rays, high-resolution computed tomography scans,* etc.). *In this scenario, the assessment would likely not be awarded due to the discrepancy between the stated diagnosis and its supporting evidence.*
Support	**Antifibrotic therapies** *such as #{DRUG BRAND NAME} and #{DRUG BRAND NAME} have been found to potentially slow down the decline in lung function in individuals with pulmonary fibrosis, particularly those suffering from its most prevalent form, idiopathic pulmonary fibrosis (#IPF).*
Support	*A recent publication in this @{CHANNEL} highlights the complexities of Idiopathic Pulmonary Fibrosis. The authors note the importance of adhering to six key principles of care outlined by {ASSOCIATION}. These guidelines emphasize the need for comprehensive management strategies. Furthermore, the article draws attention to the resources provided by* **@{ASSOCIATION}***, which offers vital support to those affected by this condition*.
Support	*I’ve been experiencing severe respiratory issues and chronic discomfort, which led to a diagnosis of pulmonary fibrosis, a condition I’ve struggled with for several years. Last year, I came across a narrative about* **{ORGANIZATION}** *claiming to cure various ailments such as herpes virus, COPD, ALS, Hepatitis, HIV, HPV, and pulmonary fibrosis. Intrigued by their claims, I reached out to this group, who provided me with a natural remedy that I used for several months. To my surprise, the treatment alleviated my symptoms entirely, and subsequent tests revealed that my IPF had vanished without any residual effects*.

Instances of these concepts within the examples are highlighted in bold for emphasis.

The potential of zero-shot classification for extracting information about diverse interest groups within the PFDD context has been previously recognized (Karmalkar et al., 2023). Building upon this, our work integrates question-answering techniques to pinpoint precise information within extended posts. This methodology parallels traditional information extraction pipelines, which often employ text embeddings like Sentence Transformers (Reimers and Gurevych, 2019). Such pipelines typically utilize these embeddings to identify a set of relevant documents which is semantically most similar to a search query (akin to our hypothesis), for example, by calculating the cosine similarity. Subsequently, they apply question-answering to the most relevant documents to extract specific information. A parallel can also be drawn with retrieval-augmented generation frameworks (Lewis et al., 2020), which have recently gained prominence alongside the rise of Large Language Models (LLM). Both these approaches and ours share the goal of deriving answers from extensive document collections. However, the key difference lies in our use of an extractive approach, aiming to extract answers from each individual document or even sentence, as opposed to abstractive approaches that generate a single response from a large corpus. While extractive results could be obtained solely through question-answering, prefacing this with zero-shot classification effectively reduces the incidence of false positives.

### 4.3 Relation extraction

**Figure 8:**
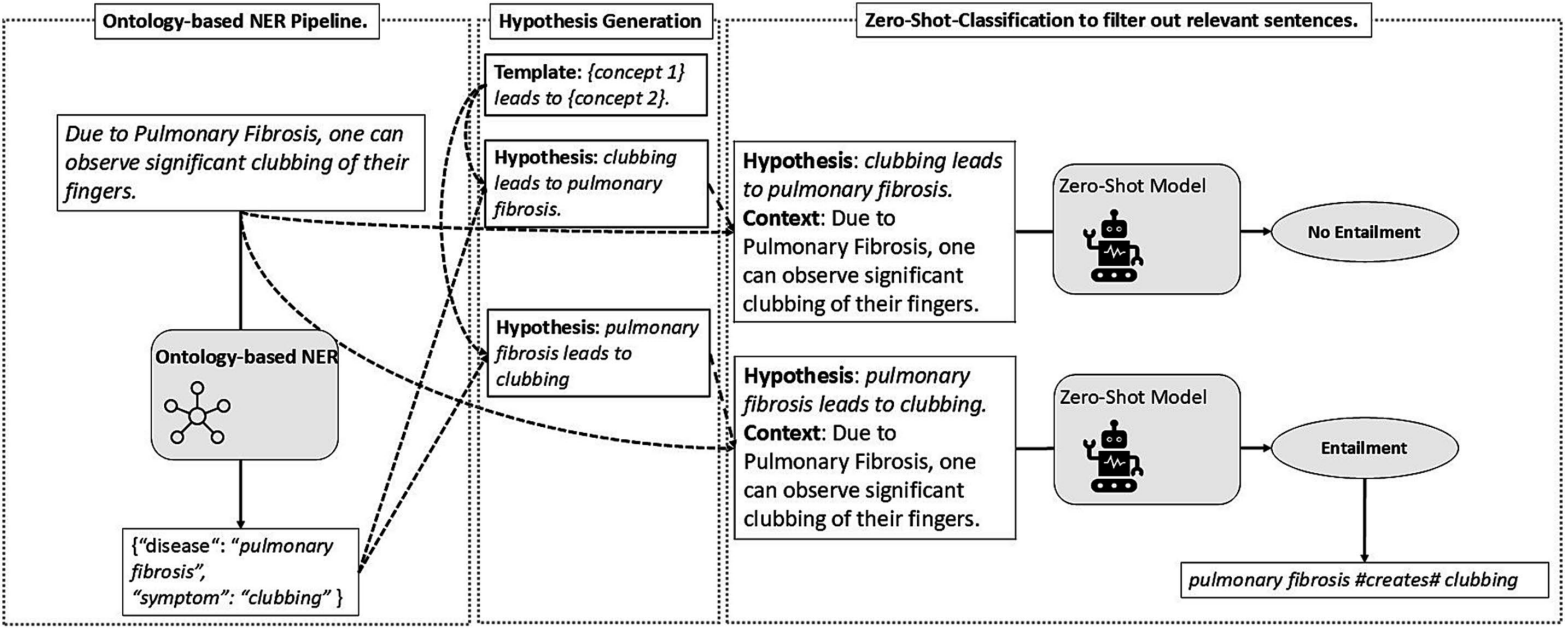
Illustration of the relation extraction pipeline by using ontology-based named entity recognition and zero-shot-classification.

Once the concepts are extracted, the relation extraction pipeline is initiated. Similar to Obamuyide and Vlachos (2019) and Hu et al. (2022), we treated relation extraction as a natural language inference task while incorporating general heuristics, as depicted in Figure 8. With a list of concepts present in a sentence at our disposal, we generate several template-based hypotheses by filling missing slots with the text spans of each concept. Each hypothesis template corresponds to a specific relation. For example, the hypothesis template *{concept 1} leads to {concept 2}* can be used for the causal *creates*-relation. Filling the slots with the spans of the concepts in the sentences generates meaningful hypotheses, which, together with the sentences themselves acting as the premise, are fed to a natural language inference model. For instance, given the sentence “*I have severe clubbing of my fingers due to my Pulmonary Fibrosis and I sometimes do not read what I have tweeted”* and the concepts *pulmonary fibrosis* (disease) and *clubbing* (symptom), we generate two hypotheses: *pulmonary fibrosis leads to clubbing* and *clubbing leads to pulmonary fibrosis*. Generating two hypotheses with swapped positions of the concept spans is essential to determine the direction of the relation. As a binary classification task, the natural language inference model subsequently returns a score between 0 and 1 for both hypotheses. The higher the score, the more likely the hypothesis holds true in the given context. This process is repeated with each hypothesis template for each relation. Since only one relation can hold between two concepts, the highest-scoring hypothesis at the end of the iteration represents the relation between the concepts if the score reaches a predefined threshold – determined through trial and error. An overview of the hypotheses used, i.e., templates, for relation extraction, is provided in Supplementary Table 4.

To expedite the process and avoid false positives, we applied ontological knowledge to eliminate hypotheses, i.e., relations, that cannot exist between two concepts. For instance, when given the concepts *idiopathic pulmonary fibrosis* (disease) and *yesterday* (date), the template *{concept 1} leads to {concept 2}* can be disregarded, as a disease cannot cause a point in time, or vice versa.

As highlighted in Section 4.2, the semantic difference between some relations is very subtle. For instance, in the example sentence above, both the *creates* and the *is associated with* relation can be applicable between the above-mentioned concepts *pulmonary fibrosis* (disease) and *clubbing* (symptom). However, for our use case, a clear distinction between these two relations is crucial. This can pose a challenge for natural language inference models trained on general-purpose datasets that may not align with our specific use cases and definitions of relations. To address this, we created a list of trigger words for the micro-relations *ameliorates*, *creates*, and *exacerbates* (*cf.* Supplementary Table 2) to further improve the results. As a final step, if the relation returned by the previously described process belongs to the set of micro-relations, i.e., *ameliorates*, *creates*, and *exacerbates*, we check whether any lexical triggers of the associated micro-relation occur in the sentence. If this is not the case, we change the micro-relation to the respective macro-relation. This approach follows the annotation guidelines established by Dunietz et al. (2017), which mandate the presence of cue words or connectives as a requisite for detecting causal relationships.

We conducted evaluations of several natural language inference models, specifically: “roberta-large-mnli”^10^ (NLI-RoBLarge), “covid-twitter-bert-v2-mnli”^11^ (NLI-CTB), “sileod/deberta-v3-base-tasksource-nli”^12^ (NLI-DeBBase), and “sileod/deberta-v3-large-tasksource-nli”^13^ (NLI-DeBLarge). The performance assessment was carried out using a range of thresholds: 0.1, 0.2, 0.3, 0.4, 0.5, 0.6, 0.7, 0.8, and 0.9. The results at the optimal threshold of 0.2 are presented in Table 7. We report on the relation extraction performance both with cue words (Panel A) and without cue words (Panel B). Although the natural language inference models alone demonstrate satisfactory results, incorporating cue words significantly enhances overall performance. It was observed that all natural language inference models faced challenges with the *is associated with* macro-relation, which notably benefited from the inclusion of cue words.

**Table 7:** Results of relation extraction using various natural language inference models.

(A) Results with cue words											
AM	CR	DG	EX	IAW	ITO	IUF	NoRel	ACC	M-F1	W-F1	Model
88.89	70	88.89	90.91	87.8	94.85	84.62	77.45	82.9	85.42	83.27	NLI-DeB_Base_
46.15	90.14	90.91	80	87.8	95.92	95.65	83.87	88.71	83.81	88.6	NLI- DeB_Large_
66.67	60.71	88.89	90.91	82.05	96.97	78.79	73.68	79.03	79.83	79.07	NLI-CTB
88.89	81.82	88.89	90.91	82.05	95.92	80	77.78	83.23	85.78	83.71	NLI-RoB_Large_

(B) Results without cue words											
AM	CR	DG	EX	IAW	ITO	IUF	NoRel	ACC	M-F1	W-F1	Model
94.74	72.13	88.89	90.91	0	94.85	76.29	70.54	75.48	73.54	73.57	NLI- DeB_Base_
46.15	93.15	90.91	80	0	95.92	88.89	75.83	81.29	71.36	78.8	NLI- DeB_Large_
75	60.71	88.89	90.91	0	96.97	75	68.68	73.23	69.52	71.05	NLI-CTB
94.74	85.29	88.89	90.91	0	95.92	50	68.16	72.58	71.74	69.62	NLI-RoB_Large_

The term “NoRel” refers to instances where named entities are identified in the document, yet no relationships exist between them. The F1-score is provided for each type of relation. Furthermore, for each model, we include key metrics: overall accuracy (ACC), Macro-F1 score (M-F1), and Weighted-F1 score (W-F1). The results are organized into two sections: the first table shows outcomes with the use of cue words, while the second table presents results obtained without employing cue words.

All models demonstrated a robust Macro-F1 score across each relation type. Prior efforts in relation extraction without training specific models exist. For instance, methods such as those outlined by Doan et al. (2019) often rely on dependency trees and a variety of hand-crafted rules to discern relations. We emphasize that our approach offers several benefits over traditional machine learning and rule-based models. Notably, it eliminates the need for specific annotation efforts, requiring instead a meticulously formulated set of hypothesis templates and a collection of common trigger words for each relation. However, this method has its limitations. While the performance of relation extraction can be improved to some extent by incorporating additional hypothesis templates and cue words, this increases processing time and has limitations when compared to dedicated model training, where each additional training instance is expected to contribute to better performance. Although this approach has yielded promising results in our domain-specific use case, the effectiveness of general-purpose natural language inference models on domain-specific relations still requires further investigation. To further enhance performance, it may be necessary to train specialized relation extraction models. Another potential approach would be to augment datasets for general-purpose natural language inference models with domain-specific examples, maintaining their flexibility while improving performance for domain-specific relation extraction.

Relation extraction facilitates a more fine-grained analysis. For instance, it enables the identification of specific sentences within lengthy documents that pinpoint the onset of a disease, such as IPF. Typically, this onset is denoted by a date expression. By using the posting date as a reference, we can calculate the precise number of days elapsed since the disease’s onset, provided the date expression is adequately specific. Table 8 presents various examples from patients and caregivers illustrating this. Notably, in most instances, the reported beginning of IPF aligns with its clinical diagnosis.

**Table 8:** Example sentences where either a patient or a caregiver describes the onset of IPF.

Interest group	*Sentence*	Abs. days
CG	*A family member’s diagnosis with idiopathic pulmonary fibrosis (IPF) led to a life-changing event: a successful lung transplant 2 years ago. This experience sparked a desire for proactive steps towards better health.*	730
PA	*When someone mentioned 2 years ago that I had idiopathic pulmonary fibrosis (IPF), I realized how little people knew about this condition - neither I nor anyone close to me had ever heard of it before.*	730
CG	*I am familiar with the experience - my mother received a diagnosis of idiopathic pulmonary fibrosis last year.*	365
PA	*In January, I received a diagnosis of IPF (Idiopathic Pulmonary Fibrosis).*	244
PA	*Two weeks ago I received a diagnosis of IPF.*	14
PA	*After undergoing a successful treatment, I’m currently managing idiopathic pulmonary fibrosis (IPF).*	0
PA	*I’m sharing my experience - at 25 years old, I contracted COVID-19 during the second wave when vaccines were still scarce where I lived. Unfortunately, now this led to the development of idiopathic pulmonary fibrosis (IPF).*	0
CG	*RT @{ASSOCIATION}: earlier this year a family member was diagnosed with idiopathic pulmonary fibrosis (IPF), prompting us to share their story as part of our ongoing effort to raise awareness about this condition,…*	0

## 5 Discussion and conclusion

This paper examines various aspects of SML in the context of PFDD by defining three broad research topics: (RT1) *Identification of Interest Groups*, (RT2) *Understanding Challenges*, and (RT3) *Assessing Treatments and Support Systems*. These research topics have been designed to be applicable to a wide range of diseases, and in this study, we specifically apply them to the disease IPF. The presented framework integrates multiple NLP components, such as external knowledge bases (ontologies), few-shot text classification and zero-shot text classification in the form of natural language inference, and question-answering, to properly address these research topics. By combining these components that presented framework seeks to reduce both development and annotation efforts while providing reliable results.

For RT1, we trained a classifier on a limited dataset of annotated social media posts, enabling it to accurately identify five primary interest groups: *caregivers, patients, the scientific/medical community, patient associations*, and *others*. Notably, only a minority of posts originated from either patients or caregivers. This contrasts with the typical binary categorization of *patient* versus *caregiver* in similar SML studies. Our results highlight the importance of clearly defining interest groups as well as the inclusion of additional categories for effective differentiation. The classifier, as all our components, operates at the sentence level, considering that perspectives may vary within longer posts. Future enhancements could include the analysis of surrounding sentences to better understand the overall discourse and thereby improving the classification accuracy.

For RT2 and RT3, our approach involved multiple NLP techniques. Initially, we developed a keyword matcher using various scientific ontologies to identify challenges (diseases and symptoms) and supports (drugs and treatments) faced by patients and caregivers. While this method facilitated rapid development of a named entity recognition system, its moderate precision and low recall indicate the need for improvement. Nevertheless, a co-occurrence analysis of symptoms and treatments associated with IPF provided valuable insights into the challenges and support mechanisms. Furthermore, we extracted time-related information using a fine-tuned BERT-based named entity recognition model. This enabled more detailed analysis of the challenges encountered by patients and caregivers. A key component of our framework is the heavy use of natural language inference models for zero-shot text classification. By using natural language inference, our work aligns with recent efforts to efficiently extract meaningful information from social media posts on biomedical topics through zero-shot methods (Fisher et al., 2023; Gan et al., 2023; Karmalkar et al., 2023; Kuntsche et al., 2023; Manias et al., 2023; Zhu et al., 2023). In contrast to previous work, we leverage natural language inference to tackle several tasks, including text classification and named entity recognition (*cf.* Section 4.2.3) and, more importantly, relation extraction (*cf.* Section 4.3). By formulating specific hypotheses and treating named entity recognition and relation extraction as classification tasks, we effectively detected additional and semantically more abstract named entities, such as *diagnosis, misdiagnosis*, *support*, and *need*, and relationships between various concepts. Relation extraction was particularly beneficial for detailed analysis, as evidenced by posts discussing the onset of diseases.

Last but not least, generative AI methods (including LLMs) have recently achieved significant breakthroughs across various domains and NLP tasks including text classification (Wang et al., 2023), named entity recognition (Ashok and Lipton, 2023), and relation extraction (Zhao et al., 2023). Despite their impressive performances, one major drawback of LLMs is the substantial computational resources and hardware required to run them. The presented NLP framework was developed as part of a larger project focusing on the semantic mining of IPF-related social media posts. The project required the analysis of large amounts of social media data within a reasonable timeframe. As we had to exclude API services due to the high research value of the data for PFDD, the technical equipment, in our case a Tesla P100-PCIE-16GB GPU, prevented us from leveraging LLMs at that time. Since the aforementioned task could be achieved using other NLP methods that are both accurate and faster, we chose not to include LLMs in the current study. Generative tasks, such as summary creation, would have benefited from the use of LLMs. However, their use would have introduced additional challenges, including the risk of hallucinations, which requires further testing. Nevertheless, recent advances – such as model quantization (Jin et al., 2024) and the availability of smaller LLMs with robust performance – open up the possibility for future integration of LLMs into our framework for SML related to PFDD.

## Data Availability

The datasets presented in this article are not readily available because they are proprietary and subject to specific licenses. Requests to access the datasets should be directed to CV, c.devuono@chiesi.com.

## Footnotes

^1^Throughout the paper, we will use the name *Twitter* instead of *X*.

^2^ https://spacy.io/

^3^ https://github.com/joke2k/faker

^4^ https://inception-project.github.io/

^5^ https://huggingface.co/sentence-transformers/all-mpnet-base-v2

^6^ https://spacy.io/api/matcher

^7^ https://huggingface.co/satyaalmasian/temporal_tagger_BERT_tokenclassifier

^8^ https://huggingface.co/sileod/deberta-v3-base-tasksource-nli

^9^ https://huggingface.co/deepset/roberta-large-squad2

^10^ https://huggingface.co/roberta-large-mnli

^11^ https://huggingface.co/digitalepidemiologylab/covid-twitter-bert-v2-mnli

^12^ https://huggingface.co/sileod/deberta-v3-base-tasksource-nli

^13^ https://huggingface.co/sileod/deberta-v3-large-tasksource-nli
