# Supplementary material for: One size fits all: Enhanced zero-shot text classification for patient listening on social media
Source: Front Artif Intell. 2025 Feb 11;7:1397470. doi: 10.3389/frai.2024.1397470 (PMC11850375; doi:10.3389/frai.2024.1397470)
Supplement: Supplementary file 1 [file Data_Sheet_1.PDF]

Supplementary Material

1 Supplementary Figures and Tables

1.1 Supplementary Tables

Table 1: Queries used to extract IPF-related posts.

Indication	Query
Idiopathic Pulmonary Fibrosis (IPF)	"Idiopathic Pulmonary Fibrosis" OR "Idiopathic Lung Fibrosis" OR "Lung Fibrosis" OR "Pulmonary Fibrosis" OR "Airway Fibrosis" OR "idiopathic interstitial lung disease" OR "Interstitial lung disease" OR "IPF"

Table 2: Overview of utilized ontologies: Due to the absence of dedicated ontologies for treatments, we employed Wikidata. We retrieved all terms classified under the superclasses *therapy* and *medical procedure*. Upon manual review, the compiled list was found to produce favorable outcomes.

Name	Concept	URL
Mondo Disease Ontology (Mondo)	Disease	https://mondo.monarchinitiative.org/
Disease Ontology (DO)	Disease	https://disease-ontology.org/
International Classification of Diseases 11th Revision (ICD10)	Disease	https://icd.who.int/en
Drug Ontology (DrOn)	Drug	https://bioportal.bioontology.org/ontologies/DRON
Symptom Ontology	Symptom	https://www.ebi.ac.uk/ols/ontologies/symp
Wikidata	Treatment	https://www.wikidata.org/wiki/Wikidata:Main_Page

Table 3: Overview of hypotheses, i.e., templates, for classifying sentences based on whether they pertain to the topics 'need,' 'support,' 'diagnosis,' or 'misdiagnosis'. Additionally, we provide a set of questions for each topic to facilitate evidence extraction from the sentences.

category	hypothesis	questions
need	*Someone needs something because of a medical condition.*	*What does someone need?*
need	*Something is needed because of a medical condition.*	*What is needed?*
need	*Someone needs something.*	*What is needed?*

need	*Someone expresses a need because of a medical condition.*	*What need does someone express?*
need	*Someone expresses a need.*	*What need does someone express?*
need	*Someone wishes something.*	*What does someone wish?*
need	*Someone wishes something because of a medical condition.*	*What does someone wish?*
support	*A patient receives support from something or someone.*	*From whom does the patient receive support?*<br>*What kind of support does the patient receive?*<br>*What kind of support is given?*
support	*Someone receives support from something or someone.*	*From whom does the person receive support?*<br>*What kind of support does the person receive?*<br>*What kind of support is given?*
support	*A patient receives help from something or someone.*	*From whom does the patient receive help?*<br>*What kind of help does the patient receive?*<br>*What kind of help is given?*
support	*Someone receives help from something or someone.*	*From whom does the person receive help?*<br>*What kind of help does the person receive?*<br>*What kind of help is given?*
diagnosis	*{concept_1} was diagnosed.*	
misdiagnosis	*{concept_1} was misdiagnosed.*	

Table 4: Overview of hypotheses, i.e., templates, for identifying the relation between two concepts.

**relation**	**hypothesis**
creates	*{concept_1} creates {concept_2}.*
creates	*{concept_1} is a side effect of {concept_2}.*
is_used_for	*{concept_1} is a treatment for {concept_2}.*
is_used_for	*{concept_1} is used for {concept_2}.*
is_used_for	*{concept_1} is used as a treatment for {concept_2}.*
is_used_for	*{concept_1} is used as a therapy for {concept_2}.*
is_used_for	*{concept_1} treats {concept_2}.*
ameliorates	*{concept_1} improves {concept_2}.*
ameliorates	*{concept_1} ameliorates {concept_2}.*
ameliorates	*{concept_1} decreases {concept_2}.*
ameliorates	*{concept_1} reduces {concept_2}.*
exacerbates	*{concept_1} makes {concept_2} worse.*
exacerbates	*{concept_1} exacerbates {concept_2}.*
exacerbates	*{concept_1} increases {concept_2}.*
ameliorates	*{concept_1} helps against {concept_2}.*
is_time_of	*a person waited {concept_1} for the diagnosis {concept_2}.*

is_time_of	*{concept_1} a person got the diagnosis {concept_2}.*
is_time_of	*a person got the diagnosis on {concept_2} {concept_1}.*
is_time_of	*{concept_1} a person got the diagnosis on {concept_2}.*
is_time_of	*a person takes {concept_2} {concept_1}.*
is_time_of	*a person took {concept_2} {concept_1}.*
is_time_of	*a person got {concept_2} on {concept_1}.*
is_time_of	*a person got {concept_2} {concept_1}.*
is_time_of	*a person was infected with {concept_2} on {concept_1}.*
is_time_of	*a person has {concept_2} since {concept_1}.*
is_time_of	*a person suffers from {concept_2} for {concept_1}.*
is_time_of	*a person has been taking {concept_2} for {concept_1}.*
is_time_of	*a person took {concept_2} since {concept_1}.*
diagnoses	*{concept_1} diagnoses {concept_2}.*
diagnoses	*{concept_1} is used to diagnose {concept_2}.*
diagnoses	*{concept_1} detects {concept_2}.*
diagnoses	*{concept_1} shows {concept_2}.*
diagnoses	*{concept_1} reveals {concept_2}.*
diagnoses	*{concept_1} can diagnose {concept_2}.*
diagnoses	*{concept_1} can help to diagnose {concept_2}.*
diagnoses	*{concept_1} diagnoses {concept_2}.*
diagnoses	*{concept_1} is used to diagnose {concept_2}.*
diagnoses	*{concept_1} detects {concept_2}.*
is_used_for	*{concept_1} is used for {concept_2}.*
is_used_for	*{concept_1} treats {concept_2}.*

Table 5: This list presents cue words associated with three specific relations: *ameliorates*, *creates*, and *exacerbates*. It is notable that there is an overlap among cue words across these relations. The context determines the applicability of certain trigger words, as some may be relevant to multiple relations.

**Relation**	**Cue words**
ameliorates	*ablate, abolish, abrogate, ameliorate, at bay, attenu, attenuate, benefici, benefit, benefit by, benefit from, block, bring down, control, decline, decrease, defens, degrade, diminish, effect, eliminate, eradicate, favorable, govern, help, improve, induce, inhibit, inhibitor, kill, knock down, knockdown, lower, mediate, minimize, modul, optimize, protect, prevent,*

	promise, promising, protect, reduc, reduce, regulate, reliev, repress, rescue, restore, reverse, revert, slow, slow down, stabilize, stimulate, stop, success, suppress, unblock, lessen
creates	*accelerate, activate, advance, affect, alter, amplify, as a consequence, as a consequence of, as a result, as a result of, because, because of, bring about, bring on, cause, cause by, cause for, cause of, causes for, causes of, co-express, coadministrate, complicate, consequently, contribute to, convert, create, deregulate, derive from, drive, due, due to, effect, effect of, elevate, elicit, enforce, enhance, enrich, entail, escalate, evoke, exert, expand, express, extend, for this reason alone, fuse, generate, give rise to, govern, grow, impact, impair, implicate in, in consequence of , increase, induce, infect, inject, intensify, lead to, maximize, modulate, originate from, originate in, over-express, owing to, predispose, produce, progress, proliferate, promote, provoke, re-express, reactivate, reason, reason for, reason of, reasons for, reasons of, regain, remove, replicate, reproduce, result, result from, result in, sensitize, stem from, stimulate, the reason for, the reason of, trigger, trigger off, unblock, up-regulate*
exacerbates	*accelerate, advance, affect, amplify, co-express, complicate, contribute to, deregulate, drive, effect, elevate, elicit, enforce, enhance, enrich, escalate, exacerbate, expand, extend, grow, impair, increase, intensify, maximize, modulate, over-express, progress, proliferate, promote, re-express, reactivate, regulate, replicate, reproduce, stimulate, worse, worsens, grow, impair, regain*

1.2 Supplementary Figures

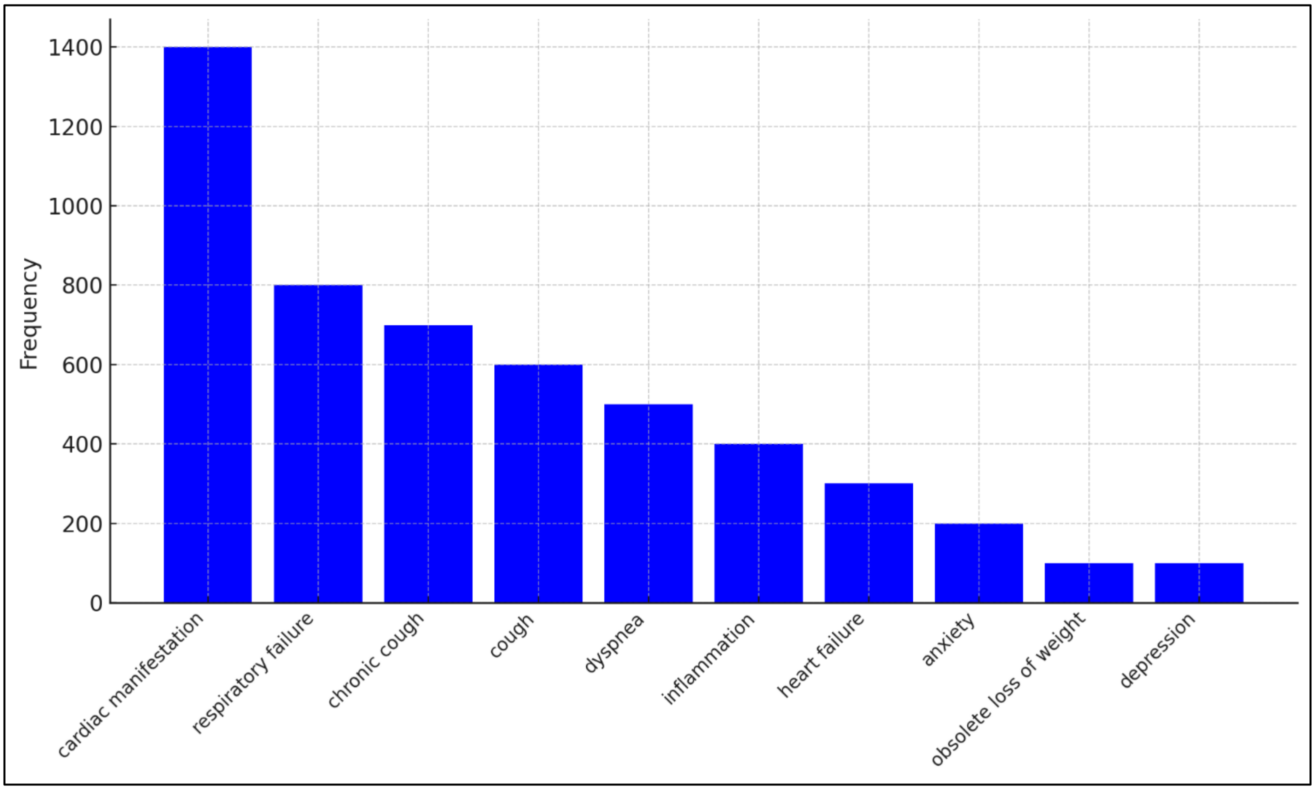

Figure 1: Frequency of the top 10 symptoms that co-occur with the term “(idiopathic) pulmonary fibrosis” or its synonyms in the same sentence. The diagrams illustrate the normalized names of named entities, instead of their textual forms. For example, 'idiopathic pulmonary fibrosis' frequently abbreviated as 'IPF,' is represented in its normalized form as 'idiopathic pulmonary fibrosis.' Such variations are standardized by ontologies.

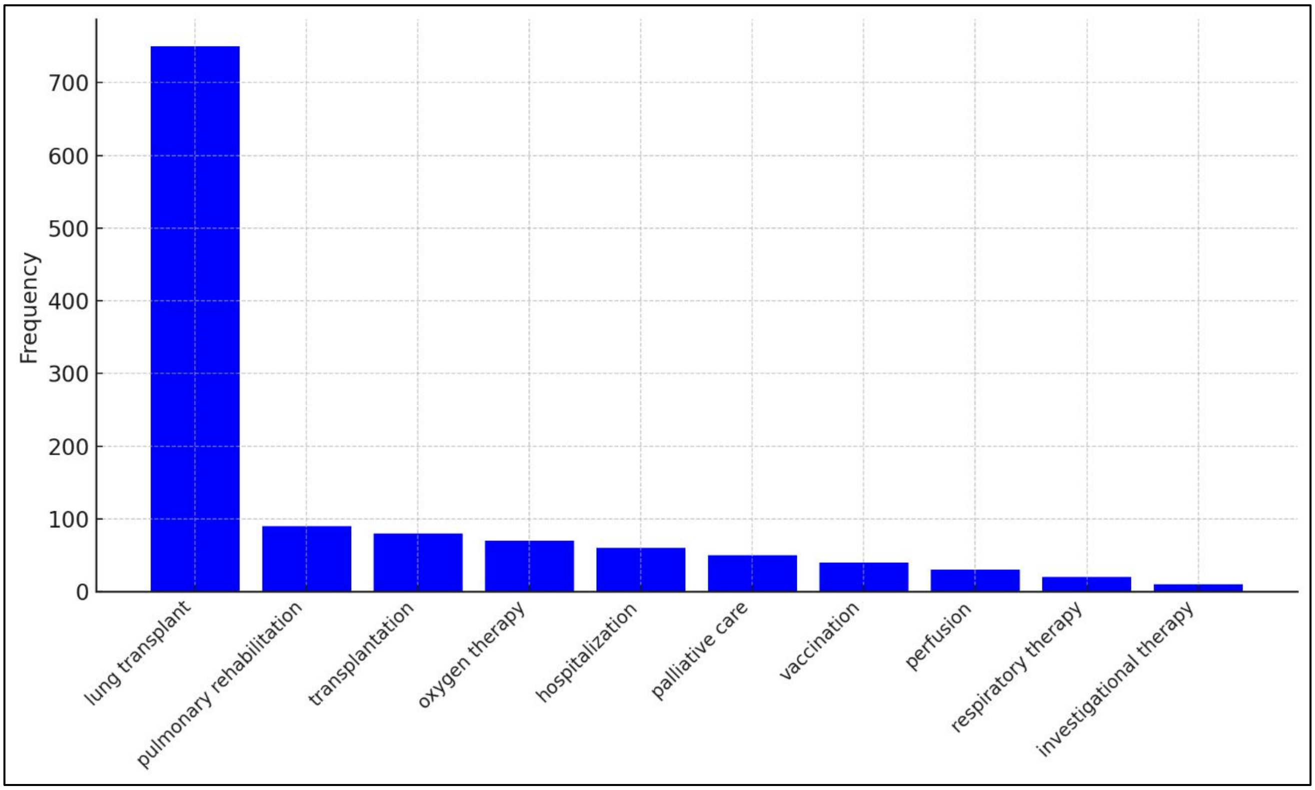

Figure 2: Frequency of the top 10 treatments that co-occur with the term "(idiopathic) pulmonary fibrosis" or its synonyms in the same sentence. The diagrams illustrate the normalized names of named entities, instead of their textual forms. For example, 'idiopathic pulmonary fibrosis' frequently abbreviated as 'IPF,' is represented in its normalized form as 'idiopathic pulmonary fibrosis.' Such variations are standardized by ontologies.

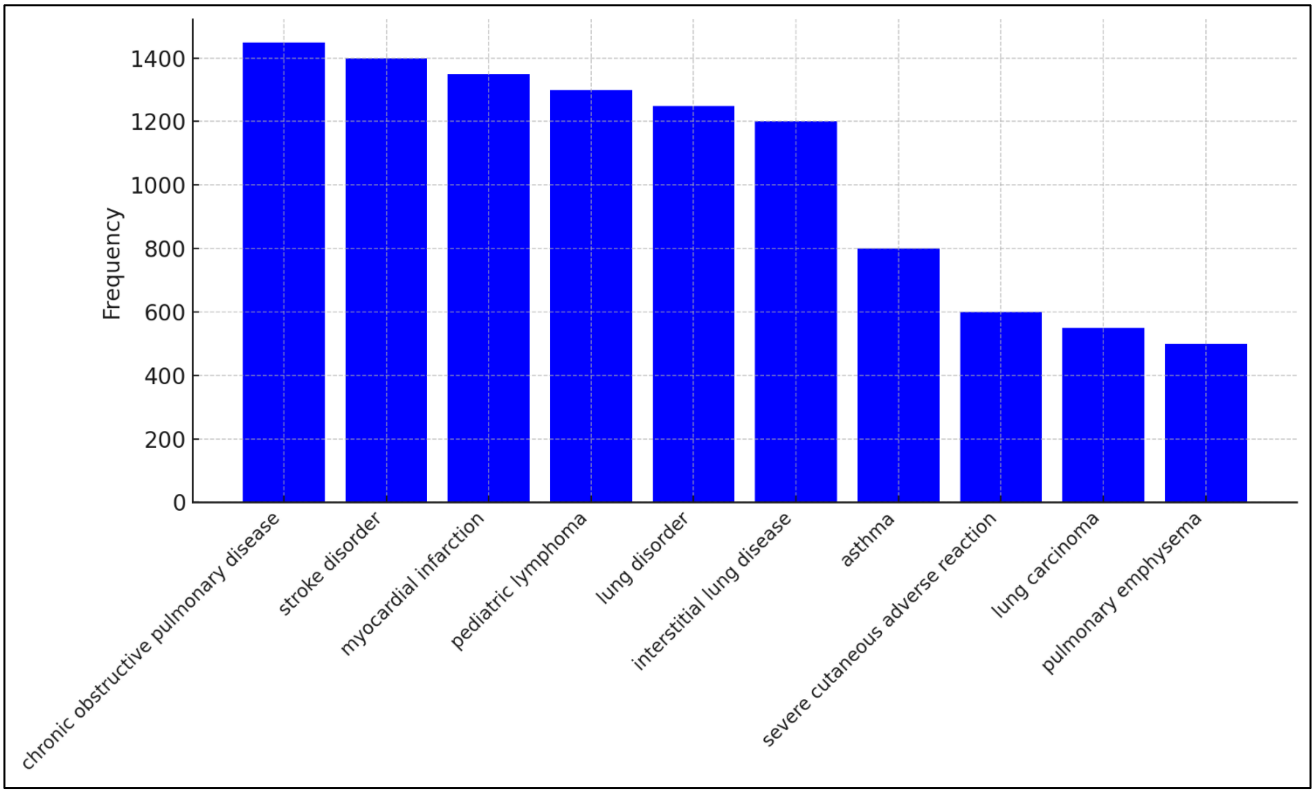

Figure 3: Frequency of the top 10 diseases that co-occur with the term "(idiopathic) pulmonary fibrosis" or its synonyms in the same sentence. The diagrams illustrate the normalized names of named entities, instead of their textual forms. For example, 'idiopathic pulmonary fibrosis' frequently abbreviated as 'IPF,' is represented in its normalized form as 'idiopathic pulmonary fibrosis.' Such variations are standardized by ontologies.
